# Competence-based curriculum development for general practice in Germany: a stepwise peer-based approach instead of reinventing the wheel

**DOI:** 10.1186/1756-0500-6-314

**Published:** 2013-08-09

**Authors:** Jost Steinhaeuser, Jean-François Chenot, Marco Roos, Thomas Ledig, Stefanie Joos

**Affiliations:** 1 Department of General Practice and Health Services Research, University Hospital Heidelberg, Voßstraße 2, Heidelberg, D-69115, Germany; 2 Institut for Community Medicine, Section Family Medicine, University Medicine Greifswald, Greifswald, Germany; 3 German College of General Practitioners and Family Physicians Chairs of the Section Postgraduate Specialty Training, Frankfurt, Germany; 4 Competence Centre General Practice Baden-Wuerttemberg, Heidelberg, Germany

**Keywords:** Curriculum development, General practice, Physician shortage, CanMEDS

## Abstract

**Background:**

Improving postgraduate medical training is one important step to attract more medical students into general practice. Keeping pace with international developments moving to competence-based curricula for general practice training, the aim of this project was to develop and implement such a curriculum in Germany.

**Methods:**

A five-step, peer-based method was used for the curriculum development process including panel testing and a “test version” of the curriculum for the pilot implementation phase. The CanMEDS framework served as a basis for a new German competence-based curriculum in general practice training. Four curricula from European countries and Canada were reviewed and, following required cultural adaptions, key strengths from these were integrated. For the CanMEDS “medical expertise” element of the curriculum, the WONCA ICPC-2 classification of patient’s “reason for encounters” was also integrated.

**Results:**

Altogether, 37 participants were involved in the development process representing 12 different federal states in Germany, and including an expert advisor from Denmark. An official “test version” of the curriculum consisting of three parts: medical expertise, additional competencies and medical procedures was established. A system of self-assessment for trainees was integrated into the curriculum using a traffic light scale. Since March 2012, the curriculum has been made freely available online as a “test version”. In 2014, an evaluation is planned using feedback from users of the test model as a further stage of the implementation process.

**Conclusions:**

The first German competence-based curriculum for general practice training has been developed using a pragmatic peer controlled approach and implementation is being trialed with a “test version” of the curriculum. This model project and its peer-based methodology may support competence-based curriculum development for other medical specialties both inside and outside Germany.

## Background

Although there is some variation in the scope of general practice between different countries, there are several common aspects e.g. first contact access for most health problems, long-term person-centered care and coordination of care [1]. The World Health Organization therefore generally recommends a specific educational curriculum for primary care [2].

According to Kern, the curriculum building processes include six steps [3]: Analyze which type of physician is the correct one for the health care system. Which are the demands for a curriculum? What are the learning goals and how can they get reached? How can changes be implemented, followed by feedback and evaluation of the curriculum?

Previous studies show that General Practitioners (GPs) in Germany are dissatisfied with the latest developments of the health care system and with the lack of structure and realistic learning goals within the postgraduate medical education [4-8].

In recent decades, a noticeable shift in focus in medical education has occurred, moving from knowledge acquisition towards knowledge application [9,10]. Furthermore, competence-based programs have become well-established in medical education curricula. Reasons for the widespread implementation of competence-based curricula include: they are believed to be a more reliable way to ensure that every graduate is prepared for practice and they have been shown to be effective in identifying areas of strength and needs in learners and allow teachers to assess appropriate learning outcomes [11,12]. Additionally there is evidence that competence-based training helps to improve resident performance and patient safety [13].

One of the most well-established competency models used in (postgraduate) medical education today is the Canadian CanMEDS framework [14]. This framework describes seven key roles highlighting the range of competencies in a physician’s professional performance: the medical expert being central, along with the communicator, the collaborator, the health advocate, the manager, the scholar and the professional.

Under current German law, the State Medical Associations of Germany (*Landesärztekammern*) are in charge of setting postgraduate educational standards in Germany. This includes general practice training, which as it presently stands, is a five year training program, encompassing a “volume- and time-based curriculum”. The time-based component requires five years of training, after which an application for examination as a GP can be made. The volume-based component is structured around a catalogue of skills and procedures that have to be accomplished and confirmed by the trainer (e.g. 150 ultrasounds of the thyroid gland) [15].

Skills and procedures currently required in general practice training are not determined by a general practice professional or academic body but are set by the State Medical Associations of Germany, based on generalized standards without a primary care or general practice specialty focus. A major disadvantage with this current general practice training curriculum is the lack of formalized structure and an element of chance related to practice experience in terms of what an individual trainee learns [4-6,16]. Switzerland has reported similar challenges related to vocational medical training in general practice [17]. Therefore the integration of a “competency” focus in contemporary general practice is believed of major importance for two reasons. Firstly, to ensure that trainees are systematically prepared with the knowledge, skills and attributes needed to provide competent patient-centered care in today’s evolving and complex healthcare services and, secondly, to make the specialty more attractive as a means of responding to workforce shortages of general practitioners. These factors created the drive to optimize general practice training as a vital element in building a sustainable future GP workforce in Germany.

The lack of a competence-based curriculum, however, is not a specific problem to general practice medical training, but relevant for all medical specialties in Germany. For instance German surgeons, have worked on a competence-based curriculum for their specialty [18]. The aim of this report is to describe the development of the first German competence-based curriculum for general practice covering a standard set of competencies from a GP point of view.

## Methods

The competency-based curriculum development process was initiated by a core group of 6 GPs and 2 GP trainees. The infrastructure of the Competence Centre General Practice Baden-Wuerttemberg facilitated the coordination of study related activities and made rooms available for meetings. As this initiative progressed, the process was strongly supported by the German College of General Practitioners and Family Physicians (DEGAM), in particular, through the recruitment of participants in the peer review process [19]. The first planning meeting was held in March 2010 moderated by an experienced curriculum specialist from Denmark. At this meeting, the first steps for the curriculum development were defined, namely to use a stepwise, peer-based approach and to recruit further participants.

### Stepwise peer based approach

**Figure 1:**
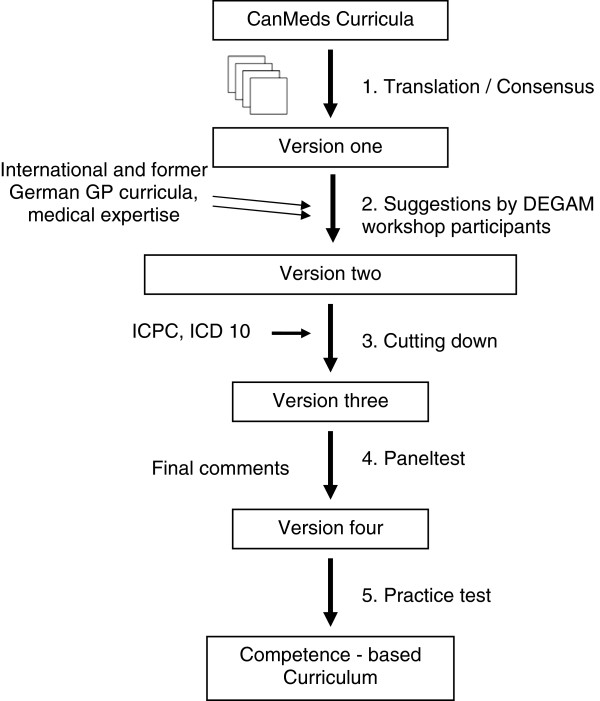
The five steps of peer based curriculum development.

The general methodological approach was pragmatically driven. It was decided that the German curriculum should be developed on the basis of existing competency-based curricula using a stepwise, peer based approach including a panel test. Changes suggested during the peer review process, were incorporated into the curriculum by members of the core group only after further discussion. The stages of this curriculum development were developed by the group as part of an ongoing process, balancing quality and feasibility aspects, and resulted in a five-step framework described in detail in the results section. The stepwise approach for curriculum development is described in detail in Figure 1.

### Recruitment

Recruitment of GPs and GP trainees during the development of the curriculum was primarily done via two national online forums for GPs, one mainly used by trainees and newly qualified GPs (http://www.jungeallgemeinmedizin.de) and an online chatroom accessible for both GPs and GP trainees (Listserverallgemeinmedizin: http://www.listserv.dfn.de/archives/allgmed-l.html).

### Ethics approval

The Heidelberg University Medical Faculty Ethics Committee informed us in the context of a former qualitative study with GPs, that approval by an ethics committee was not necessary as the study did not involve patients and their personal data (personal communication to last author 17/07/05).

## Results

### Participants

12 of the 17 federal states in Germany were represented, with 37 participants involved in the process. An external advisor came from Denmark. Participants were on average 48 (29-60) years old and mostly male (65%). The majority of the participants (86%) were members of the DEGAM. Sociodemographical data of the participants are shown in Table 1.

**Table 1:** Participant sociodemographic* (n = 37)

**Age mean (range)**	**48,3 (29-60 years)**
Sex (n)	Female: 13 Male: 24
GP specialists (n)	30
GP trainer (n)	21
Non-physician (n)	1
Medical student (n)	1
Trainees/Member of the JADe^1^ (n)	10
Member of the DEGAM^2^ (n)	32
Member of the HÄV^3^ (n)	16
Position in a Medical Association (n)	7

*Multiple answers were possible.

^1^JADe: The German Working Group for Newly Qualified and Future General Practitioners.

^2^DEGAM: German College of General Practitioners and Family Physicians.

^3^HÄV: Professional Association of Family Physicians and General Practitioners in Germany.

### Curriculum structure

Due to the cultural adaption by the peers, the competence-based general practice curriculum consists of three parts: part one for medical expertise, part two for additional competencies and part three for medical procedures.

1. **Selection and translation**

A core decision made at the first planning meeting was to base the curriculum on the CanMEDS. Other frameworks, such as the educational agenda of EURACT [20] were considered, but the structure of the CanMEDS framework was preferred and considered to be easier to transfer to a German context. A further key reason behind the choice of the CanMEDS framework is that there is an ongoing project by the Association for Medical Education and the Association of Medical Faculties in Germany rebuilding the undergraduate medical education curriculum on the basis of the CanMEDS framework [21]. Accessibility was an additional consideration as the CanMEDS based curricula of Canada and Denmark were easily available [22,23].

The CanMEDS framework was translated from English into German. Each role of the CanMEDS framework was translated by two separate groups. Since participants came from different federal states, they formed smaller regional working groups. Their translations were compared at a consensus meeting of members of the core group. Afterwards, the translation results were compiled into a draft of the curriculum (“Version one”).

2. **DEGAM congress**

In 2010, “Version one” was presented at the national congress of the DEGAM in Dresden at a workshop [24]. During this workshop, more than 30 GPs from all over Germany discussed the results and provided feedback. Suggestions were gathered to support the “trialing phase” of the implementation process. On the basis of the workshop discussions, the following criteria and steps were agreed among the core group:

○ The project was adopted as a project of the DEGAM section postgraduate specialty training.

○ All competencies listed were to denote the minimum baseline, not the maximum.

○ The medical expertise section was to be revised integrating the frequency and importance of patients’ “reasons for encounter” in Germany

○ Other competencies (outside medical expertise) were to be revised

○ An additional chapter of diagnostic-therapeutic procedures performed in German GP practices was to be added

○ Consideration of other curricula was to be undertaken, namely the Swiss general practice curriculum, the curriculum of the former German Democratic Republic (DDR), the content of the former compulsory GP course after the German reunification, the experiences of the participants and the “Canon GP” [25-28].

A group of six GPs and one trainee from three different federal states met to rework the “medical expert” section considering the above mentioned points. In addition, every participant of this group revised one competence of the curriculum. The resulting “version two” of the curriculum had more than 70 pages, including 43 pages for medical expertise. “Version two” was rated by peers for missing topics and feasibility.

3. **Cutting down process**

One of the main points peers suggested was to cut down the draft curriculum by at least 50%. Equally, it needed to be ensured that no important competency area was lost during this review process. Therefore, the International Classification of Primary Care (ICPC–2), a classification developed for general practice by the International Classification Committee of the World Organization of National Colleges, Academies and Academic Associations of General Practitioners/Family Physicians (WONCA), was used to frame the medical expertise part of the curriculum. ICPC-2 allows classification of the patient’s reason for encounter, the problems/diagnosis managed, interventions, and the arrangement of these data in order of an episode-of-care structure [29]. Additionally, the reasons for encounter were verified by using the 50 most common GP diagnoses in Germany [30].

**Figure 2:**
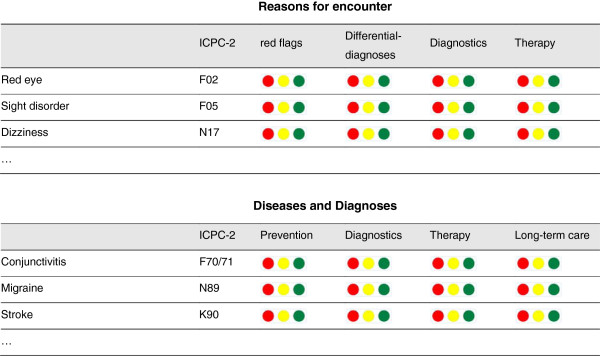
Example of the medical expertise part of the competence - based curriculum.

This cutting down process allowed baseline competencies to be identified, which a trainee would need to achieve to become a GP in Germany. At this stage, it was agreed to define the contents of GP specialty training along the requirements of actual daily work. Nevertheless, there was discussion about the depth to which these baseline competencies should be mastered. As a result, a system of self-assessment was integrated into “Version three”. Both the individual trainee and the GP trainer are able to carry out formative assessments using a traffic light scale to identify areas of strengths and weakness. The traffic lights range from red: *“I have no competence in this area and therefore I feel unsafe*”, yellow: *“I gained some competence in this area but I need to improve to feel safe”* to green: *“I consider myself competent in this area and feel safe”.* A second reason to introduce this traffic light assessment scale was to facilitate a culture of providing feedback from the trainer to the trainee. For this reason, a form that can be used for feedback discussions was added to the appendix of the curriculum document. Figure 2 gives an example of the general structure of the medical expertise part.

4. **Panel-test**

“Version three” underwent a panel test in November 2011. All “panel test” participants were asked to rate every single chapter with school grades (1 = very good to 6 = insufficient). All chapters reaching a rating of 3 or better were regarded as achieving a “pass” through the panel test. Additionally, participants were asked to comment if they identified missing or inappropriate parts of the curriculum. 15 participants were involved in the panel test. From these 15, one participant did not give grades to every single chapter of the “version three” but gave overall feedback. All chapters passed the first round of the panel test with a result of 2.3 on average from the remaining 14 participants. Furthermore, additional written comments from the participants were considered and where appropriate incorporated in the next revision. The revised “version three” again was sent to all participants for final review.

5. **Practice test**

Following feedback on the revised “Version three”, “Version four” was produced and accepted as the “test version”. Since March 2012, the “Version four” curriculum document has been made freely available online as the “practice test version”:

(http://www.kompetenzzentrum-allgemeinmedizin.de/public/curriculum.shtml).

To download the curriculum an email address has to be provided. After two years, an evaluation process is planned, which involves asking every person downloading the curriculum about his/her experience with the curriculum and the feasibility of implementation. This feedback will be used to build the “Version five”, the finalized competence-based curriculum for general practice training in Germany. To date, the curriculum has been downloaded 428 times, including from colleagues from African and Scandinavian countries. We are aware that copies of the curriculum are distributed freely and assume that this number is underestimating the distribution of the curriculum.

## Discussion

For the first time in Germany, a competence-based GP curriculum has been developed. Instead of completely reinventing the wheel, a peer-based approach was used adapting existing international best practice models to national needs. To our knowledge, this is the first time such a method has been used for curriculum building.

Peer reviewers came mainly from general practice and were predominantly members of the DEGAM. The support of two formal general practice networks, the Competence Centre for General Practice Baden-Wuerttemberg and the DEGAM was very important for the success of this process. This was possible because members of the core group were actively involved in these networks.

It was decided to use the CanMEDS framework as a foundation. Compared to other frameworks, the structure of the CanMEDS seemed easiest to understand and to transfer to a German context. Translation of the framework was a necessary, but straightforward process. However, all competencies required cultural adaptions. In particular, the health advocate role was challenging to adapt to the German health care setting. Although procedures are competencies in a most basic sense, participants in the peer review process considered it to be a matter of importance, that a skill based section should be added to the curriculum. Therefore an additional chapter on diagnostic-therapeutic procedures was added to ensure the learning needs of trainees were effectively addressed in this area of practice development.

In addition to publication, a further strategy supporting uptake of this model competence-based general practice training curriculum in Germany is that it is readily available online and easy to use. Moreover, there is official backing from the DEGAM and support from the *Junge Allgemeinmedizin Deutschland [The German Working Group for Newly Qualified and Future General Practitioners]*, a newly established forum supporting and informing trainees and newly qualified GPs in Germany and the *Deutscher Hausärzteverband [Professional Association of Family Physicians and General Practitioners in Germany]*, the largest organization aiming to support interests of primary care physicians in Germany.

### Strengths and limitations

For this process of a competence-based curriculum building a pragmatic method was taken (adapting existing international best practice models to the Germany context) as neither funding nor a mandate from responsible regulators was available. Indeed, the medical specialty of General Practice is still in the process of gaining influence and recognition in Germany. Nevertheless, great care was taken to involve participants with a genuine interest in the improvement of general practice training without raising conflicts of interest in regard to political bodies. A strength of this process is that peer trainers and trainees were actively involved in the drafting and consensus process, which increases the likelihood of “ownership” and acceptance of the curriculum. Results regarding the feasibility of implementation will be available following the planned evaluation in 2014.

### Next steps

The “test version” curriculum will be subject to a formal evaluation in 2014. Following finalization, on-going review and re-evaluation will be regularly carried out to ensure the curriculum remains responsive to changing needs in the primary care context. Furthermore, once the project integrating the CanMEDS framework into the German undergraduate medical education curriculum is finished, alignment work will be carried out to ensure a natural transition from undergraduate training to postgraduate general practice training [21]. Finally, in a positive new development, the responsible regulators at federal level (*Bundesärztekammer*) have started a project building competency based curricula for all medical specialties [31]. Also due to the preliminary work reported here, two of the authors have been invited to participate at this project to work on the official future GP competency based curriculum.

## Conclusions

The lack of a competence-based curriculum is not a specific problem to general practice medical training, but relevant for all medical specialties in Germany. The first German competence-based curriculum for general practice training has been developed using a pragmatic peer controlled approach and implementation is being trialed with a “test version” of the curriculum. This model project and its peer-based methodology may support competence-based curriculum development for other medical specialties both inside and outside Germany.

## Availability of supporting data

The German competence-based curriculum for general practice can be downloaded in German language after registration here: http://www.kompetenzzentrum-allgemeinmedizin.de/public/curriculum.shtml.

## Competing interests

Authors disclose any competing interests.

## Authors’ contributions

All authors have made substantial contributions to conception and design of the study. JS did coordinate the whole process of curriculum building. JS, JFC and SJ have been involved in drafting the manuscript. MR and TL did critically revise the manuscript. All authors have read and approved the final manuscript.
